# Antimicrobial Activity of Hybrid Nanomaterials Based on Star and Linear Polymers of *N*,*N*′-Dimethylaminoethyl Methacrylate with In Situ Produced Silver Nanoparticles

**DOI:** 10.3390/ma13133037

**Published:** 2020-07-07

**Authors:** Paulina Teper, Anna Sotirova, Violeta Mitova, Natalia Oleszko-Torbus, Alicja Utrata-Wesołek, Neli Koseva, Agnieszka Kowalczuk, Barbara Mendrek

**Affiliations:** 1 Centre of Polymer and Carbon Materials, Polish Academy of Sciences, M. Curie-Sklodowskiej 34, 41-819 Zabrze, Poland; pteper@cmpw-pan.edu.pl (P.T.); noleszko@cmpw-pan.edu.pl (N.O.-T.); autrata@cmpw-pan.edu.pl (A.U.-W.); akowalczuk@cmpw-pan.edu.pl (A.K.); 2 Stephan Angeloff Institute of Microbiology, Bulgarian Academy of Sciences, Georgi Bonchev Str. 26, 1113 Sofia, Bulgaria; anna@microbio.bas.bg; 3 Institute of Polymers, Bulgarian Academy of Sciences, Georgi Bonchev Str. Bl. 103A, 1113 Sofia, Bulgaria; mitova@polymer.bas.bg (V.M.); koseva@polymer.bas.bg (N.K.)

**Keywords:** star polymers, antimicrobial activity, poly(*N*,*N*′-dimethylaminoethyl methacrylate), silver nanoparticles

## Abstract

Well-defined linear and multi-arm star polymer structures were used as the templates for in situ synthesis and stabilization of silver nanoparticles (AgNPs). This approach led to hybrid nanomaterials with high stability and antibacterial activity to both Gram-positive and Gram-negative bacterial strains. The ecologically friendly so called “green” synthesis of nanomaterials was performed through AgNPs preparation in the aqueous solutions of star and linear poly(*N*,*N*′-dimethylaminoethyl methacrylate)s (PDMAEMAs); the process was followed with time. The size, shape, and zeta potential of the obtained hybrids were determined. To our knowledge, this is the first time that the antibacterial activity of PDMAEMA hybrid nanomaterial against *Bacillus subtilis*, *Escherichia coli* and *Pseudomonas aeruginosa* was investigated and assessed by minimum inhibitory concentration (MIC) and minimum biocidal concentration (MBC). Completely quaternized with ethyl bromide, star and linear PDMAEMAs were used in comparative biological tests. The modification of the polymers with in situ-formed AgNPs increased the antibacterial properties against all studied strains of bacteria by several times in comparison to non-modified polymers and quaternized polymers. These results yield novel nanohybrid materials that can be useful for applications in medicine and biology.

## 1. Introduction

In the face of an epidemiological threat that has recently overtaken the world (SARS-CoV-2), it is important to know the effects of materials against many pathogens. For example, reduced antibiotic activity due to an increase of bacterial resistance is one of the biggest problems facing medicine. Therefore, it is important to find new solutions to this issue.

Silver has been known as an antimicrobial agent since antiquity. In recent years, the silver nanoparticles (AgNPs) have attracted considerable attention because of their unique antimicrobial activity among other properties [1,2,3,4]. It has been reported that metal colloidal nanoparticles exhibit the size and shape-dependent properties therefore, the precise control over those parameters becomes critical for their applications [5,6]. The size and shape of AgNPs also greatly influence the antimicrobial properties. If the dispersions of nanoparticles are not stable or they aggregate to larger structures, their antibacterial activity is reduced [7].

Certain polymers may serve as macromolecular support to enhance the long-term stability of silver nanoparticles [7]. Most often silver nanoparticles are formed by chemical reduction by using sodium borohydride, for example, and stabilized by a polymer [8,9] or a surfactant [7].

Poly(*N*,*N*′-(dimethylamino)ethyl methacrylate) (PDMAEMA) is a good cationic polymer for stabilizing AgNPs and exhibits biocidal properties by itself [10,11,12]. Functional amine groups of these polymers are often additionally quaternized to enhance their bactericidal activity [11,13,14,15] because they are capable of interacting with negatively charged bacterial cell surface structures. Alkyl halides are most commonly used for the quaternization reaction [11,12]. The advantage of PDMAEMA is also that the presence of amine groups could be used to produce AgNPs from silver ions without any additional chemical reductant, i.e., applying a “green” synthesis approach.

Environmentally friendly chemical syntheses are becoming more desirable. “Green” synthesis of AgNPs using the *N*,*N*′-dimethylaminoethyl methacrylate homopolymer has been described in very few papers [4,16,17,18]. Shvedchenko et al. [16] reported the formation of AgNPs using linear PDMAEMA, poly[2-deoxy-2-methacrylamido-D-glucose], and their copolymers. The authors proposed a mechanism of AgNPs formation based on the ratio of the particle diameter to the average number of reducing sites per monomer, depending on the composition of the polymer. The authors consider that formed silver atoms stay close to the reducing centers; when the reaction is completed, the polymer chains entwine the formed nanoparticles, preventing its further growth [16]. Sun et al. [17] found similar mechanisms, but with more stages, for 4-arm star and linear PDMAEMA during the reduction of silver ions to AgNPs in water solutions. The authors claimed that the formation of silver nanoparticles comprises three steps. First, silver ions are trapped on the PDMAEMA surface and are reduced to form silver clusters. In the next step, adsorption of PDMAEMA on silver clusters takes place with further reduction of silver ions and accumulation of silver clusters. The last step involves the growth of silver particles and their stabilization by PDMAEMA [17]. Polymeric micelles of star-shaped copolymers composed of ε-caprolactone, *N*,*N*′-(dimethylamino)ethyl methacrylate and oligo(ethylene glycol) monomethyl ether methacrylate units were used by Huang et al. [18] for the preparation of AgNPs and then were used as a catalyst. However, in all of the above-mentioned research, the antibacterial properties of the obtained hybrid materials were not investigated.

Recently, Lin et al. [4] used micelles of linear and 4-arm triblock copolymers of DMAEMA, 2-hydroxyethyl methacrylate, and poly(ethylene glycol) monomethyl ether methacrylate (PEGMA) for AgNPs formation and investigated antimicrobial activity against one bacterial strain *Escherichia coli*; the inhibition of bacterial growth was dependent on the concentration of hybrid materials. The higher concentrations were needed to stop bacterial growth in the case of AgNPs obtained in the presence of micelles formed from linear copolymers, than for the star polymer micelles [4].

Studies of how hybrid materials based on silver nanoparticles and DMAEMA homopolymers of different topologies (linear, multiarm star) act on a broad range of bacterial strains are missing. The above examples show that these studies are unsystematic.

In this work, we have undertaken such research, and designed antimicrobial polymeric materials with incorporated silver nanoparticles based on linear and star homopolymers of DMAEMA. The choice of polymer was dictated by the presence of a larger number of amine groups than in copolymers.

For this purpose, we applied well-defined linear and star macromolecules. The star consisted of 28 poly(*N*,*N*′-dimethylaminoethyl methacrylate) arms and a hyperbranched poly(arylene oxindole) core. Polymers with such a high number of arms have never been explored before for AgNPs formation, and it should be expected that the unimolecular “core-shell” structure of the stars should strongly stabilize formed AgNPs, thus preventing their unwanted aggregation. The novelty of this work was to determine the impact of DMAEMA homopolymer topology with many amine groups on the effective formation of silver nanoparticles. We also determined the influence of the obtained hybrid materials on the antibacterial properties against various bacterial strains.

Here, the in situ synthesis of the AgNPs in the presence of these macromolecules and detailed characterization of the properties of the resulting hybrid metal-polymer systems is presented. The antimicrobial activities of the obtained hybrid materials are demonstrated and compared with quaternized star and linear PDMAEMA, as well as with non-modified polymers.

In this study, model bacterial strains from different genera were used; these cause damages to industrial manufacture, agriculture and human health—*Bacillus subtilis 168* is a model strain of Gram-positive bacteria, *Escherichia coli W 1655* and *Pseudomonas aeruginosa 1390* are representatives of Gram-negative bacteria and also are important opportunistic pathogens.

The systematic research and comparative study of the influence of polymer topologies (star or linear) and structure modification (in situ-formed AgNPs or quaternization) on the antibacterial properties enabled us to create a library of data on antibacterial properties of different PDMAEMA structures and to choose the most efficient PDMAEMA-based system.

## 2. Materials and Methods

### 2.1. Materials

1,2-Dichlorobenzene (99%), 1,1,4,7,10,10-hexamethyltriethylenetetramine (HMTETA, 97%), 2-bromopropionitrile (97%), copper (I) bromide (CuBr, 99.999%), copper (II) bromide (CuBr_2_, 99%), p-xylene (≥99%) and ethyl bromide (98%) were purchased from Sigma Aldrich (Poznan, Poland) and used as received. *N*,*N*′-dimethylaminoethyl methacrylate (DMAEMA, 98%) was purchased from Sigma Aldrich and purified by distillation prior to use. An ion exchanger DOWEX MARATHON MSC was purchased from Sigma Aldrich and transformed into H^+^ using 1.6 mol/L HNO_3_. Acetone (99.5%) and silver nitrate (AgNO_3_, 99.9%) were purchased from POCH (Gliwice, Poland) and used as received. Tetrahydrofuran (THF, pure p.a.) was purchased from POCH and purified by distillation prior to use. A SpectraPor membrane with MWCO 1000 g/mol was purchased from Roth (Karlsruhe, Germany). Phosphate buffer saline (PBS) was purchased from PAA Laboratories GmbH (Oberosterreich, Austria). Mueller Hinton Broth (MHB) was purchased from HiMedia (Mumbai, India).

### 2.2. Synthesis of N,N′-Dimethylaminoethyl Methacrylate Star and Linear Polymers

Atom transfer radical polymerization (ATRP) was used for the synthesis of *N*,*N*′-dimethylaminoethyl methacrylate star [19], and linear [20] polymers. In brief, the star synthesis was performed using hyperbranched poly(arylene oxindole) (PArOx) as a macroinitiator (28 bromoester groups, M_n_ = 21,000 g/mol); for the linear polymer, 2-bromopropionitrile was used. The catalytic complex consisted of CuBr and HMTETA (ligand) was used, for stars, additionally, CuBr_2_ was added. Polymerizations were carried out in 1,2-dichlorobenzene. After the desired monomer conversion was obtained, the THF was added to the reaction mixture and the solution was passed through a column with Dowex-MSC-1 ion exchange resin (Sigma-Aldrich, Steinheim, Germany) to remove the copper. Next, the solution was dialyzed against methanol and then against water (SpectraPor membrane with MWCO 1000 g/mol), and dried by lyophilization.

### 2.3. In-Situ-Produced Silver Nanoparticles by N,N′-Dimethylaminoethyl Methacrylate Star and Linear Polymers

AgNO_3_ solution (1 mmol/L) was added dropwise to linear or star poly(*N*,*N*′-dimethylaminoethyl methacrylate) (PDMAEMA) aqueous solution (c = 1 mg/mL) and stirred. The molar ratio of DMAEMA units to AgNO_3_ (N/Ag) was 10:1. The reaction was conducted at room temperature, at pH close to 7, and was followed over time using UV-Vis, dynamic light scattering (DLS), Zetasizer and transmission electron microscopy images (TEM).

### 2.4. Quaternization of Linear and Star PDMAEMA in the Solution

Linear and star PDMAEMA were quaternized according to the procedure described in [12]. Briefly, for this purpose, 15 × 10^−3^ g (2 × 10^−2^ mol of amine groups, sample S-PDMAEMA, L-PDMAEMA, Table 1) linear or star polymer was placed in flask and dissolved in 10 mL of acetone. Then, 3.3 g (3 × 10^−2^ mol, 2.2 mL) of ethyl bromide was added ([ethyl bromide]:[amine groups] in a molar ratio 1.5:1) to the solution. The flask was immersed in an oil bath at 40 °C and kept on a magnetic stirrer for 24 h. The reaction was performed under atmospheric conditions. After the required reaction time, the quaternized PDMAEMA salt (QPDMAEMA) precipitated out of the solution and the mixture became cloudy. The QPDMAEMA was left to sediment. Then, the solvent was decanted and the quaternized polymer was washed several times with fresh acetone (20 mL each). The obtained quaternized polymers were dialyzed against deionized water for 2 days (Roth membrane, regenerated cellulose, MWCO 1000 g/mol) and dried by lyophilization to obtained white powder.

### 2.5. Culture Media and Growth Conditions

#### 2.5.1. Microorganisms

The bacterial strains used in this study were *Pseudomonas aeruginosa 1390*, *Bacillus subtilis 168* and *Escherichia coli W 1655* (National Bank of Industrial Microorganisms and Cell Cultures, Sofia, Bulgaria). The cultures were maintained at 4 °C on Bacto agar (Difco, MP Biomedicals, Eschwege Germany) slants and were transferred monthly.

The bacterial strains were grown overnight in Mueller Hinton Broth (MHB) (HiMedia) at 37 °C with stirring at 200 rpm.

#### 2.5.2. Preparation and Storage of Resazurin

Resazurin (Resazurin Sodium Salt, Sigma Aldrich) was prepared at a concentration 0.02%, vortexed, filter sterilized (0.22 µm filter) and stored at 4 °C for a maximum of 2 weeks after preparation.

#### 2.5.3. Preparation of Standardized Inoculum

The inocula were prepared in accordance with the Clinical Laboratory Standards Institute (CSLI) recommendation. The optical density value (OD)_600_ was adjusted to the equivalent of 10^8^ colony-forming units/mL (CFU/mL). The viability graph was used to calculate the actual number of colony-forming units for each microorganism.

#### 2.5.4. Preparation of 96 Well-Plates for Testing Reagents

Plates were prepared under aseptic conditions. Reagents were dissolved in water and filtered (0.22 µm filter, Millipore, Merck, Burlington, MA, USA). All the wells of plates were filled with 50 µL of Mueller Hinton Broth (MHB). The tested concentrations of the different reagents were achieved through double serial dilutions by initial transfer of 50 µL test material from the first row to the subsequent wells in the next row of the same column. Resazurin solution (30 µL) was added to each well. Finally, 10 µL from the bacterial suspension was added to each well to achieve a final concentration of 5 × 10^6^ CFU/mL. The plates were incubated in temperature-controlled incubator at 37 °C for 24 h. After incubation, the concentration of inhibitors in columns with no color change was taken as the minimum inhibitory concentration (MIC) value.

All experiments were performed in triplicate and the average values were calculated. Mean values are given with standard deviations of ≤10%.

The controls in the plates were: (a) bacteria + MHB + resazurin, (b) test agent in serial dilution + MHB + indicator and (c) bacteria + MHB

The minimum biocidal concentration (MBC) was determined when there was no colony growth from the directly plated contents of wells with concentrations higher than the MIC value on inhibitor-free MHB agar plates.

### 2.6. Methods

Gas chromatography (GC) was used to establish monomer conversion. Measurements were carried out on a Varian 3400 chromatograph.

NMR spectra were recorded using a Bruker Ultrashield 600 spectrometer (600 MHz for ^1^H, Billerica, MA, USA). The resonances are presented in ppm and referenced to the tetramethylsilane (TMS) peak.

Gel permeation chromatography with multiangle laser light scattering detection (GPC-MALLS, Malvern Panalytical Ltd., Malvern, UK) was used for the determination of the molar mass and molar mass distributions of the linear and star PDMAEMA. Analyses were performed in DMF containing 5 mmol/L of lithium bromide, at 45 °C with a nominal flow rate of 1 mL/min. The column set contained GRAM columns from Polymer Standard Service (PSS): a guard + 100 Å + 1000 Å + 3000 Å was used. Differential refractive index detector (Δn-2010 RI WGE Dr. Bures, Berlin, Germany) and a multiangle laser light scattering detector (DAWN HELEOS from Wyatt Technologies, Santa Barbara, CA, USA) were used in the system. The results were evaluated with ASTRA 5 software (Wyatt Technologies, Santa Barbara, CA, USA).

The refractive index increment (*dn*/*dc*) of the star was calculated from Equation (1):

(1) $$\frac{dn}{dc}=w_{PArOx}{\left(\frac{dn}{dc}\right)}_{PArOx}+w_{DMAEMA}{\left(\frac{dn}{dc}\right)}_{PDMAEMA},$$

where *w_PArOx_* is the weight ratio of the core, *w_DMAEMA_* is the weight ratio of DMAEMA in the star polymer, (*dn/dc*) PArOx is the refractive index increment of the PArOx core and (*dn*/*dc*) *PDMAEMA* is the refractive index increment of the PDMAEMA. The refractive index increments of PArOx (*dn*/*dc* = 0.149 mL/g) and linear PDMAEMA (*dn*/*dc* = 0.056 mL/g) were independently measured in DMF using a SEC-3010 *dn*/*dc* WGE Dr. Bures differential refractive index detector. These values were used for calculating the refractive index increment of the star PDMAEMA (sample S-PDMAEMA, Table 1) according to Equation (1).

UV-vis absorption spectra were recorded on a Jasco V-530 spectrophotometer (Tokyo, Japan). The spectra were collected in the range of 300–900 nm.

Dynamic light scattering (DLS) measurements were carried out on a Brookhaven BI-200 goniometer with a digital autocorrelator (BI-9000 AT, Brookhaven Instruments, New York, NY, USA) and vertically polarized laser light (Brookhaven Instruments, New York, NY, USA) operating at 35 mW and *λ* = 637 nm. The autocorrelation functions were examined using the constrained regularized algorithm CONTIN. The measurements were made at 25 °C and a 90° angle. Before measurements, the samples were passed through 0.2 µm membrane filters (Graphic Controls, DIA-Nielsen, Düren, Germany).

Zeta potential measurements were performed in triplicate in disposable folded capillary cells on a Zetasizer Nano ZS 90 (Malvern Instruments, Malvern, UK). The zeta potential (ζ) was calculated from the electrophoretic mobility, *u*, employing the Helmholtz–Smoluchowski Equation (2):

(2) $$u=\frac{\varepsilon \zeta }{\eta },$$

where *ε* is the dielectric constant of the solvent and *η* is the viscosity of the solvent.

Transmission electron microscopy images (TEM) were obtained using a Tecnai F20 TWIN microscope (FEI Company, Hillsboro, OR, USA). The microscope was equipped with a field emission gun operating at an acceleration voltage of 200 kV. Images were recorded on an Eagle 4k HS camera (FEI Company, USA) and processed with TIA software (FEI Company, USA). 

Atomic absorption spectroscopy (200 Series AA, Agilent Technologies, Santa Clara, CA, USA) was used to establish the concentration of the silver ions left in the solution of star and linear polymers after AgNPs formation. For this purpose, the colloidal solutions of polymer and AgNPs were centrifuged (15000 RCF, HERMLE Z32HK, HERMLE Labortechnik GmbH, Wehingen, Germany). The obtained supernatants were appropriately diluted and analyzed for Ag^+^ concentration.

## 3. Results and Discussion

### 3.1. Linear and Star poly(N,N′-Dimethylaminoethyl Methacrylate)s for AgNPs Preparation

**Scheme 1:**
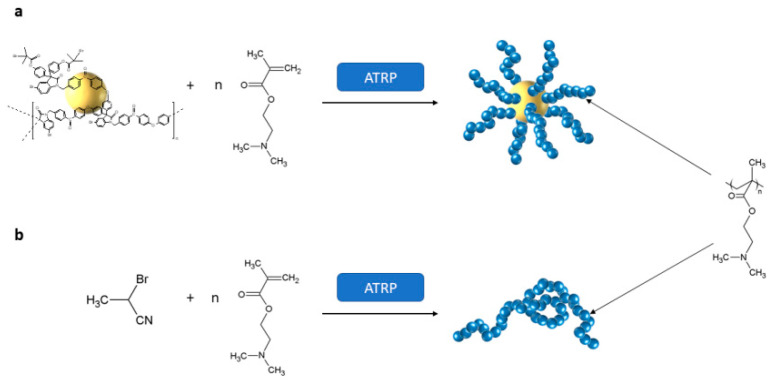
The synthesis of (**a**) star and (**b**) linear poly(*N*,*N*′-dimethylaminoethyl methacrylate)s via ATRP.

Linear and star PDMAEMA were obtained via ATRP using CuBr and HMTETA as catalytic complexes. For linear polymers, 2-bromopropionitrile as initiator was used [20]; for the star synthesis, hyperbranched poly(arylene oxindole) (PArOx) was applied as macroinitiator [21]. The schematic representation of *N*,*N*′-dimethylaminoethyl methacrylate ATRP leading to the assumed structures is presented in Scheme 1.

The ^1^H NMR spectra show the structure of the obtained polymers (Figure S1, Supporting Materials). The measurements were performed in chloroform, which is a good solvent for both studied topologies of PDMAEMA. 

The absolute molar masses and molar mass distribution of the synthesized polymers were estimated using GPC-MALLS, while the monomer conversion was followed using gas chromatography (GC) (Table 1).

**Figure 1:**
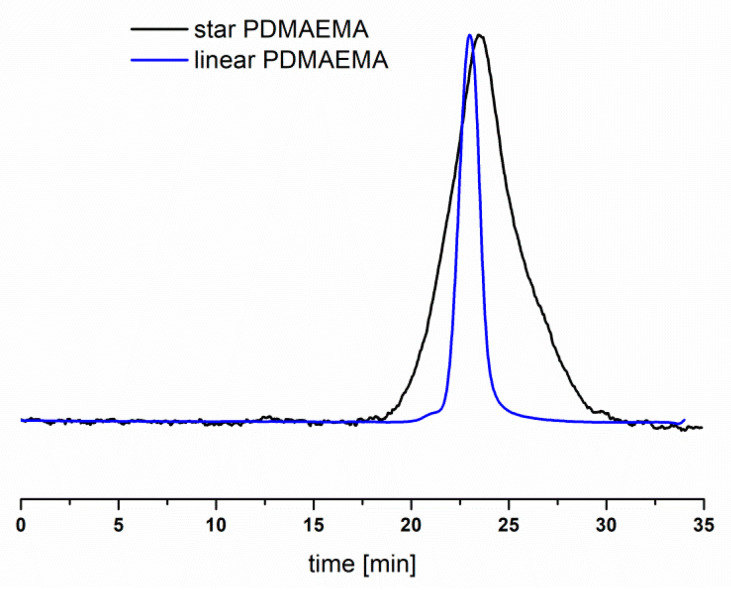
Chromatograms of star and linear PDMAEMA (DMF, 1 mL/min).

Chromatograms (detector RI response) of the synthesized polymers were shown in Figure 1.

As the result, two polymers were obtained with molar masses close to 100,000 g/mol; the topologies were linear (sample L-PDMAEMA, Table 1) and stars with 28 arms (sample S-PDMAEMA, Table 1).

The similar values for their molar masses allow their comparison in further studies in terms of the number of amine groups available in these macromolecules.

Based on the molar mass of the multiarm star and assuming equal reactivity of all PArOx initiating groups, each of the 28 arms of the star contains 25 DMAEMA units; the linear polymer is composed of seven hundred-mers of DMAEMA. The behavior of such topologies in the solution, as well as the accessibility of their functional groups, is different even though the number of amine groups is comparable. The hydrodynamic diameters of star and linear PDMAEMAs were determined using dynamic light scattering in the aqueous solution (c = 1 mg/mL) and were equal to 30 and 40 nm, respectively. The zeta potential values for both types of polymers before the synthesis of nanoparticles (c = 1 mg/mL) indicate that the stars have a higher zeta potential than the linear polymer. The zeta potential value was 35 mV for S-PDMAEMA and 15.3 mV for L-PDMAEMA. This difference is probably due to the higher density of amine groups in a spherical star PDMAEMA macromolecule compared to a linear polymer.

### 3.2. Preparation and Characterization of the Hybrid Polymeric Materials with Incorporated AgNPs

**Scheme 2:**
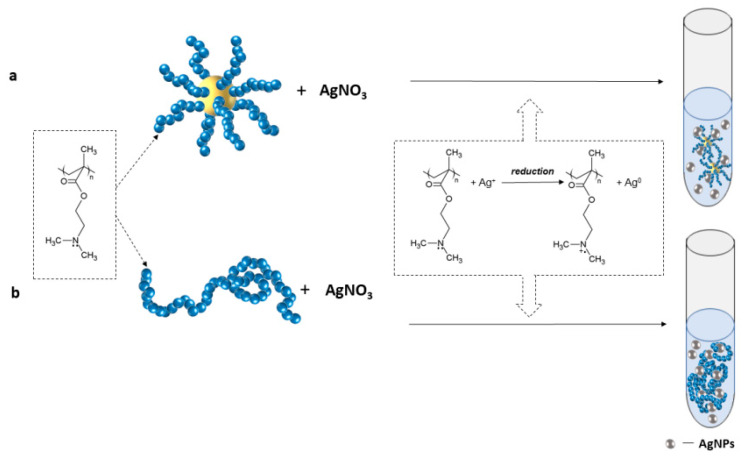
Preparation of the hybrid polymeric materials by (**a**) star polymer and (**b**) linear polymer with in situ-incorporated AgNPs.

The incorporation of AgNPs into PDMAEMA structures (linear and star) was performed via an in situ reduction reaction of silver nitrate to silver atoms using amine groups of the linear or star polymer (samples S-PDMAEMA, L-PDMAEMA, Table 1), as shown in Scheme 2. The amino-containing macromolecules were used first for the reduction of the silver ions, and second to stabilize the formed nanoparticles, preventing their undesired aggregation. In the case of polymers, the reduction time of Ag^+^ ions are longer than reduction using commonly applied agents such as NaBH_4_. The advantage of this approach is the “green” synthesis of AgNPs, conducted without additional chemical compounds, with a dual role for the polymer.

The experiments were performed by mixing the aqueous solutions of silver nitrate (c = 1 mmol/L) with a solution of star or linear PDMAEMA (c = 1 mg/mL). The mole ratio of amine groups of PDMAEMA to AgNO_3_ (N/Ag) in both polymer solutions was 10:1.

**Figure 2:**
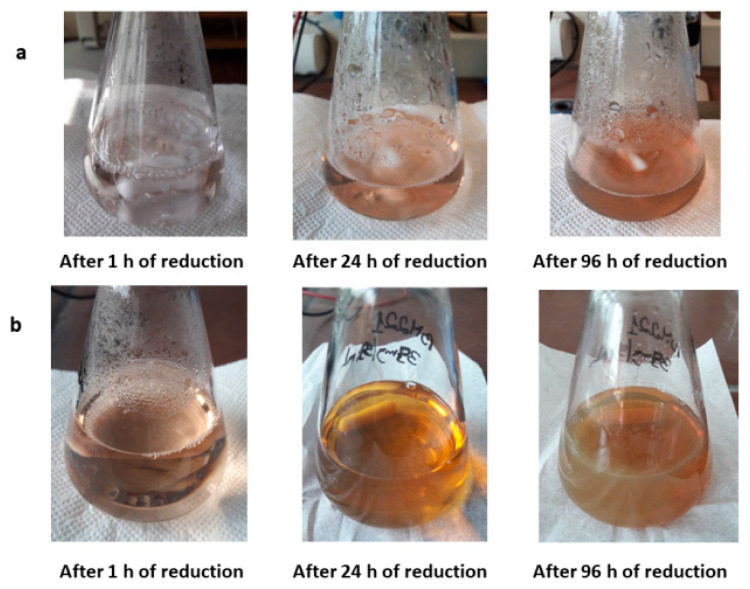
Selected time course images of AgNPs formed in aqueous solutions of (**a**) star PDMAEMA and (**b**) linear PDMAEMA.

Nanoparticle formation is accompanied by a change in the color of the solution [22]. The color of the solution changed from slightly orange after 1 h of reaction to brown after 96 h of reaction (Figure 2a,b).

The process of AgNPs formation in aqueous solutions of both types of polymers was followed over time by UV-Vis spectroscopy, dynamic light scattering (DLS) and measurements of the surface charge of obtained nanoparticles. AgNPs formation was also visualized by transmission electron microscopy, where morphology, shape and size of the nanoparticles were determined. Silver ion concentrations in the reaction mixture after the AgNPs formation was determined using atomic absorbance spectrometry (AAS).

The silver nanoparticles exhibit a characteristic size-dependent surface plasmon resonance band which was measured using UV-Vis spectroscopy [17,22,23]. The position of this band on the absorbance spectrum depends on the size of the nanoparticles. AgNPs with sizes of 5–10 nm exhibit absorbance around 400 nm. As particle size increased, a shift of absorbance to longer wavelengths (500–800 nm) was observed [22], but the aggregation of silver particles caused a decrease in the intensity of absorbance [23].

**Figure 3:**
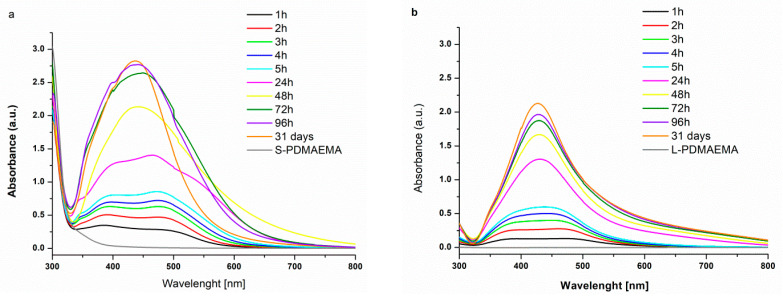
UV-Vis spectra of AgNPs formed in the aqueous solution of (**a**) star PDMAEMA (S-PDMAEMA, Table 1) and (**b**) linear PDMAEMA (L-PDMAEMA, Table 1); the polymer concentration was c = 1 mg/mL.

Figure 3 shows the UV-Vis spectra of AgNPs formed in situ in the star and linear PDMAEMA solutions during their formation process (samples described as AgNPs/S-PDMAEMA and AgNPs/L-PDMAEMA). Additionally, spectra of pure polymer solutions have been added to the graphs to show that there is no absorption from the polymer in the investigated region.

A strong increase in absorbance was observed during the course of the reaction, regardless of the type of polymer used (Figure 3), which indicates an increase of nanoparticle concentration. The presence of the band with an absorption maximum at a wavelength near 400 nm suggests that the obtained AgNPs are stable and their size is several nm.

The observed peak maximum at 440 nm in the spectra of the star polymer solution after 48 h and of the linear polymer solution after 24 h may be attributed to the surface plasmon resonance (SPR) absorption peak of spherical or nearly-spherical silver nanoparticles [4,22]. The intensity of the SPR absorption peak of nanoparticles in the solution of star PDMAEMA was higher than in the solution of linear polymer (Figure 3), which indicates that the use of star polymer enables the formation of nanoparticles in higher concentrations. Additionally, in the star polymer solution, the peak intensity after 96 h and 31 days did not change, while in the linear polymer solution, the intensity increased.

**Figure 4:**
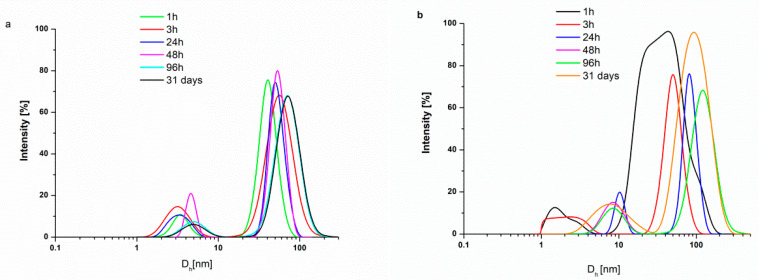
Size distributions of AgNPs formed in solutions of (**a**) star PDMAEMA and (**b**) linear PDMAEMA (DLS, θ = 90°).

Atomic absorption spectrometry was used to establish the concentration of Ag^+^ left in both the star and linear polymer solutions after 96 h of AgNPs formation. The supernatant obtained after centrifugation of AgNPs and polymers was analyzed. The silver ions concentration was 2.1 and 5.1 mg/L in the supernatant obtained from the star PDMAEMA solution and linear PDMAEMA, respectively. The initial concentration of silver ions before the reaction was 107.87 mg/L (1 mmol/L solution of AgNO_3_); therefore, in the case of linear polymer solution, this concentration was sufficient at a measurable rate, contrary to the star polymer solution. The sizes of the formed nanostructures in the solution, including AgNPs and polymers, were followed using dynamic light scattering. The average hydrodynamic diameters obtained from at least three measurements are presented in Table 2; selected size distributions are shown in Figure 4.

Regardless of the type of polymer topology and the reaction time, two modes of nanoparticles were observed for the distribution of sizes (Figure 4). The first peak, in the range of several nanometers, corresponds to the size of formed AgNPs; the second peak, in the range of several dozen nanometers, corresponds to the polymers.

As the reaction proceeded, the sizes of formed AgNPs in the solution containing star polymer did not change significantly, while the size of the polymer (30 nm) changed by nearly two times after 31 days of reaction. This increase was probably caused by the presence of metal nanoparticles, which provoked some aggregation of the star macromolecules. This behavior was more pronounced in the solution of linear PDMAEMA (Table 2), where the size of the polymer changed by more than two times after 31 days in comparison to the size of linear PDMAEMA in water (40 nm).

The sizes of formed AgNPs in linear PDMAEMA solution also varied (from 2.4 to 13.5 nm) more than in the solution of star PDMAEMA (from 4.9 to 8.4 nm). This result suggests that the formation of silver nanoparticles in the star polymer solution leads to more stable nanoparticles.

The zeta potential values determine the stability of the nanoparticles. It is accepted that a zeta potential ≥ ±30 mV indicates of colloid stability [24].

Values of zeta potential of the obtained nanostructures were positive and close to or greater than 30 mV, suggesting that they were stable in the solution (Table 2). In the case of AgNPs formed in the linear polymer solution, a significant increase (Table 2) in the zeta potential values was observed in comparison with the pure polymer solution (15.3 mV). The star polymer solution exhibited comparable zeta potential with that of the polymer with AgNPs, and a slight increase from 35 to 42.2 mV was observed after 31 days. 

**Figure 5:**
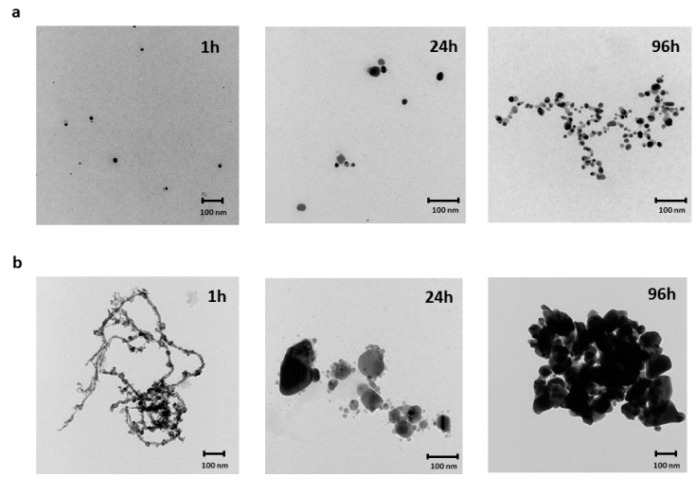
TEM images of AgNPs formed in solutions of (**a**) star PDMAEMA and (**b**) linear PDMAEMA.

TEM images were taken to determine the shape and morphology of AgNPs formed in PDMAEMA solutions (Figure 5). AgNPs formed in the solution of star PDMAEMA were mostly spherical-like particles, with the shape that remained unchanged with the reaction time; the obtained nanoparticles were separated one from another (Figure 5a). Their amount increased with the reaction time, which is coincident with the results obtained from UV-Vis measurements (Figure 3a). The linear topology of PDMAEMA chains favored the formation of different nanostructures. After 1 h of the reduction reaction, the AgNPs formed threads (Figure 5b) which, over time, reorganized into larger structures. Images taken after 24 h of reaction show AgNPs with varied sizes, which after 96 h of reaction aggregated to larger structures (Figure 5b). These results are consistent with results from DLS (Table 2).

### 3.3. Preparation of Quaternization of the Star and Linear PDMAEMA

For comparison of antibacterial activity with hybrid materials, both topologies of PDMAEMA were quaternized. This modification should increase the antibacterial activity of PDMAEMA by increasing the positive charge interaction with the bacteria membrane that facilitates its disruption.

**Figure 6:**
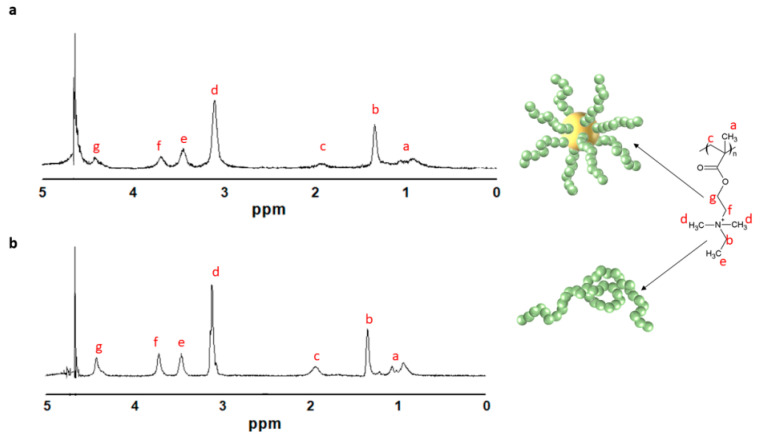
^1^H NMR (600 MHz, D_2_O) of quaternized (**a**) star PDMAEMA (**b**) linear PDMAEMA.

For this purpose, the pendant amine groups of both PDMAEMA topologies (S-PDMAEMA and L-PDMAEMA, Table 1) were quaternized with ethyl bromide in acetone, yielding QPDMAEMA bromide salts (QS-PDMAEMA and QL-PDMAEMA). The quaternization process provided 100% yields, which was verified by ^1^H NMR spectroscopy (Figure 6).

The ^1^H NMR spectra of both quaternized polymers recorded in deuterated water (Figure 6a,b) displayed peaks for α-methyl groups and methylene groups in the methacrylate backbone at δ = 0.8–1.1 ppm (a) and 1.7–2.0 ppm (c), respectively and relative to the spectrum recorded in chloroform (Figure S1). Proton signals from the methylene groups in pendant chains were found at δ = 3.6–3.8 ppm (f) and δ = 4.3–4.5 ppm (g); signals from the methyl protons of the quaternary amine group were at δ = 3.0–3.2 ppm (d). All signals were shifted compared to signals before quaternization (Figure S1), supporting that all amine groups were quaternized and the efficiency of the reaction was 100%. The ethylene group attached to the amine group gives two signals at δ = 3.4–3.5 ppm from the methyl group (e) and at δ = 1.3–1.4 ppm from the methylene group (b). The presence of these signals also confirmed the quaternization process. The spectrum of quaternized S-PDMAEMA was recorded in water, so the signals from the core were not visible due to its strongly hydrophobic character.

### 3.4. Antimicrobial Activity of Obtained Materials

The main goals of these studies was to evaluate and compare the antibacterial activity of obtained hybrid nanomaterials based on PDMAEMA structures modified with AgNPs (AgNPs/S-PDMAEMA and AgNPs/L-PDMAEMA) with quaternized linear and star PDMAEMA (QS-PDMAEMA and QL-PDMAEMA) as well as the non-quaternized form of both polymers (S-PDMAEMA and L-PDMAEMA). A resazurin-based microtiter dilution assay was used to evaluate the antimicrobial activity of all obtained polymers.

Active bacterial cells reduced the non-fluorescent resazurin (blue) to the fluorescent resorufin (pink), which was further reduced to hydroresorufin [25]. This allowed for a direct quantifiable measurement of bacterial metabolic activity.

The antimicrobial potential of all studied structures was investigated against model Gram-positive (*Bacillus subtilis*) and model Gram-negative (*Escherichia coli)* bacteria strains, which are able to cause damage to industrial manufacturing, agriculture and human health, and as well as *Pseudomonas aeruginosa*, which is an opportunistic human and plant pathogen. *P. aeruginosa* inhabits soils and aqueous environments and is highly resistant to a large number of disinfectants and antibiotics. Furthermore, *P. aeruginosa* can grow well on a variety of surfaces and is often the cause of medical device contamination and nosocomial infections [26].

Two basic parameters, the minimum inhibitory concentration (MIC) and minimum biocidal concentration (MBC) were determined for the PDMAEMA structures; the data are presented in Table 3.

Generally, MIC is the lowest concentration of the antimicrobial agent that completely inhibits visible growth of the tested organism; MBC is the lowest concentration of antimicrobial agent needed to kill 99.9% of bacteria after incubation for 24 h on inhibitor-free MHB agar plates under Clinical Laboratory Standards Institute (CSLI) standardized conditions [27]. Antimicrobial agents are usually regarded as bactericidal if the MBC/MIC ratio is ≤4 and bacteriostatic if the ratio is >4 [28].

PDMAEMA is a cationic polymer that has antibacterial activity. Both MIC and MBC values obtained for the star-shaped DMAEMA with homopolymer arms, relative to Gram-negative bacteria (*Pseudomonas aeruginosa* and *Escherichia coli*) are smaller (0.03 mg/mL) than the linear homopolymer (0.125 mg/mL). This means that the star polymer is more lethal for Gram-negative bacteria than the linear analogs. The star polymer architecture provides high polymer chain density and the arrangement of active amine groups; thus, their accessibility within the polymer is more favorable against bacteria, than in the linear topology. For *Bacillus subtilis*, both MIC and MBC values were the same for the star and linear DMAEMA homopolymers (0.03 mg/mL); so, the distribution of amine groups (polymer topology) did not affect the antibacterial properties against Gram-positive bacteria.

Functional amine groups of PDMAEMA are often quaternized to enhance their bactericidal activity. This property was the most effective for quaternized star-shaped PDMAEMA against Gram-positive *Bacillus subtilis* bacteria and for quaternized linear PDMAEMA against Gram-negative bacteria (*Pseudomonas aeruginosa* and *Escherichia coli*); MIC and MBC values halved after quaternization. Surprisingly, quaternization did not affect the antibacterial activity of the star-shaped PDMAEMA against Gram-negative bacteria or the linear PDMAEMA against Gram-positive bacteria (i.e., MIC and MBC did not change). The antibacterial activity of the quaternized star-shaped PDMAEMA was greater than that of quaternized linear PDMAEMA (i.e., MIC and MBC values for quaternized stars were two times lower than quaternary linear structures for all types of bacteria).

The modification of the polymers with in situ-formed AgNPs increased the antibacterial ability against all studied strains of bacteria by several times in comparison to non-modified polymers and quaternized structures independent of the polymer topology.

A similar relationship was observed by Lin et al [4], who investigated the antibacterial activity against *E. coli* bacteria of hybrid nanomaterials consisting of AgNPs and linear and four-arm star copolymer micelles of DMAEMA, 2-hydroxyethyl methacrylate and poly(ethylene glycol) methyl ether methacrylate micelles. The micelles formed by linear and star copolymers loaded with AgNPs exhibited higher antimicrobial activity than non-modified nanoparticles. Additionally, the concentrations of AgNPs formed in the presence of linear copolymer micelles, preventing the bacterial growth, were relatively higher than those of star copolymers micelles-AgNPs system. The authors concluded that such results might be because a bigger size of AgNPs were obtained in linear copolymer micelles than in star copolymer micelles and, therefore, lead to lower antibacterial performance because of the inefficient exposure of bacteria to AgNPs.

In our studies, there was no definitive effect of the size of obtained nanoparticles on antibacterial activity. The hybrid nanomaterials obtained using star and linear PDMAEMA showed low MIC and MBC values, which were similar to each other (Table 3). The sizes of AgNPs (Table 2, after 96 h) did not seem to influence the antibacterial properties, although the nanostructures in the hybrid star materials were smaller and exhibited higher zeta potential than in the hybrid linear materials (Table 2, after 96 h). Therefore, interactions with bacteria were a result of more than the size and surface charge of the nanomaterials.

The MIC and MBC values of non-stabilized silver nanoparticles were determined by group of Zbořil [7,29] for inter alia *E. coli* and *P. aeruginosa*. The values of minimal inhibitory concentration ranged from 1.69 µg/mL to 27 µg/mL, dependent of the reducing agent and bacteria strain and were identical with MBC values. In the case of our hybrid nanomaterials, the values are within the range reported by Zbořil [7,29]. They are also much lower than the those obtained for AgNPs formed during reduction with sodium borohydride and stabilized using linear PDMAEMA-b-PEGMA, (MIC of 0.336 mg/mL against *E. coli* and 0.393 mg/mL against *Staphylococcus aureus* [8]).

All nanomaterials in this study, as indicated by the MIC/MBC ratio, could be considered bactericidal agents. The strongest antibacterial activity was exhibited in hybrid nanomaterials of PDMAEMA and AgNPs, regardless of the polymer topology.

## 4. Conclusions

Novel hybrid nanomaterials based on star and linear PDMAEMA with in situ-produced silver nanoparticles were obtained via “green” synthesis. The well-defined structures of PDMAEMA served as a reducing and stabilizing agent. The AgNPs were obtained without using any other chemicals and under mild conditions; the process of their formation was monitored over time using UV-Vis, TEM and DLS. Based on the size, zeta potential measurements and TEM images, we concluded that AgNPs formed in star PDMAEMA solution were more stable; thus, the star structures prevented undesirable aggregation of silver nanoparticles in the solution better than the linear polymers. Antimicrobial activity of hybrid nanomaterials was compared with quaternized and non-quaternized star and linear polymers and evaluated against three strains of bacteria: *Bacillus subtilis*, *Escherichia coli* and *Pseudomonas aeruginosa.* All studied structures exhibited antibacterial activity against tested bacterial strains, but the incorporation of AgNPs into polymer structures enhanced their bactericidal activity by several times.

## Figures and Tables

**Table 1:** Polymerization conditions and molar masses of the synthesized PDMAEMA.

Sample	Topology	DMAEMA:Initiator	Monomer Conversion [%] ^a^	M_n_ [g/mol] ^b^	M_w_/M_n_^b^
S-PDMAEMA	star	2800:1	25	115,000 *	2.6
L-PDMAEMA	linear	1000:1	64	100,000	1.2

^a^ Estimated using GC; ^b^ Estimated using GPC-MALLS; * Indicates the total mass of the star polymer with the core.

**Table 2:** The hydrodynamic diameter and zeta potential of the nanostructures formed in the solution of star and linear PDMAEMA.

Time	AgNPs/S-PDMAEMA		AgNPs/L-PDMAEMA	
	D_h_ [nm]	Zeta Potential [mV]	D_h_ [nm]	Zeta Potential [mV]
1 h	4.9; 38.8	38.0	2.4; 46.4	27.6
2 h	4.8; 34.2	39.0	6.3; 46.8	27.5
3 h	3.2; 38.9	39.6	4.1; 51.0	29.5
4 h	4.7; 38.4	39.6	8.2; 58.9	31.2
5 h	3.6; 33.7	39.4	5.6; 50.0	31.2
24 h	4.2; 45.9	42.0	9.0; 64.7	35.3
48 h	4.6; 42.3	42.8	8.9; 76.0	36.8
72 h	4.9; 56.2	45.5	8.4; 76.3	39.4
96 h	5.0; 69.5	49.8	8.4; 87.9	38.8
31 days	8.4; 71.0	42.2	13.5; 92.0	39.2

**Table 3:** Determination of minimum inhibitory concentration (MIC) and minimum biocidal concentration (MBC) values and bactericidal capacity (MBC/MIC) of polymers and AgNPs-polymer nanostructures.

Sample	STRAIN								
	*Pseudomonas aeruginosa 1390*			*Escherichia coli W 1655*			*Bacillus subtilis 168*		
	MIC [mg/mL]	MBC [mg/mL]	MBC/MIC	MIC [mg/mL]	MBC [mg/mL]	MBC/MIC	MIC [mg/mL]	MBC [mg/mL]	MBC/MIC
S-PDMAEMA	0.03	0.03	1	0.03	0.03	1	0.03	0.03	1
QS-PDMAEMA ^a^	0.03	0.03	1	0.03	0.03	1	0.015	0.015	1
AgNPs/S-PDMAEMA ^b^	0.01	0.01	1	0.01	0.01	1	0.01	0.01	1
L-PDMAEMA	0.125	0.125	1	0.125	0.125	1	0.03	0.03	1
QL-PDMAEMA ^c^	0.06	0.06	1	0.06	0.06	1	0.03	0.03	1
AgNPs/L-PDMAEMA ^d^	0.01	0.01	1	0.02	0.02	1	0.01	0.01	1

^a^ quaternized star polymer; ^b^ star polymer with AgNPs; ^c^ quaternized linear polymer; ^d^ linear polymer with AgNPs.

## Supplementary Materials

The following are available online at https://www.mdpi.com/1996-1944/13/13/3037/s1, Figure S1: ^1^H NMR spectra of (a) star PDMAEMA and (b) linear PDMAEMA (CDCl_3_, 600 MHz).
