# Supplementary material for: Antimicrobial Activity of Hybrid Nanomaterials Based on Star and Linear Polymers of N,N′-Dimethylaminoethyl Methacrylate with In Situ Produced Silver Nanoparticles
Source: Materials (Basel). 2020 Jul 7;13(13):3037. doi: 10.3390/ma13133037 (PMC7372353; doi:10.3390/ma13133037)
Supplement: Supplementary file 1 [file materials-13-03037-s001.pdf]

materials

Supplementary Materials: Antimicrobial activity of hybrid nanomaterials based on star and linear polymers of N,N′-dimethylaminoethyl methacrylate with in situ produced silver nanoparticles.

Paulina Teper [1], Anna Sotirova [2], Violeta Mitova [3], Natalia Oleszko-Torbus [1], Alicja Utrata-Wesołek [1], Neli Koseva [3], Agnieszka Kowalczuk [1] and Barbara Mendrek [1,*]

[1] Centre of Polymer and Carbon Materials, Polish Academy of Sciences, M. Curie-Sklodowskiej 34, 41-819 Zabrze, Poland; pteper@cmpw-pan.edu.pl (P.T.), noleszko@cmpw-pan.edu.pl (N.O.-T.), autrata@cmpw-pan.edu.pl (A.U.-W.), akowalczuk@cmpw-pan.edu.pl (A.K.)
[2] Stephan Angeloff Institute of Microbiology, Bulgarian Academy of Sciences, Georgi Bonchev Str. 26, 1113 Sofia, Bulgaria; anna@microbio.bas.bg (A.S.)
[3] Institute of Polymers, Bulgarian Academy of Sciences, Georgi Bonchev Str. Bl. 103A, 1113 Sofia, Bulgaria; mitova@polymer.bas.bg (V.M.), koseva@polymer.bas.bg (N.K.)
* Correspondence: bmendrek@cmpw-pan.edu.pl; +48-32-271-60

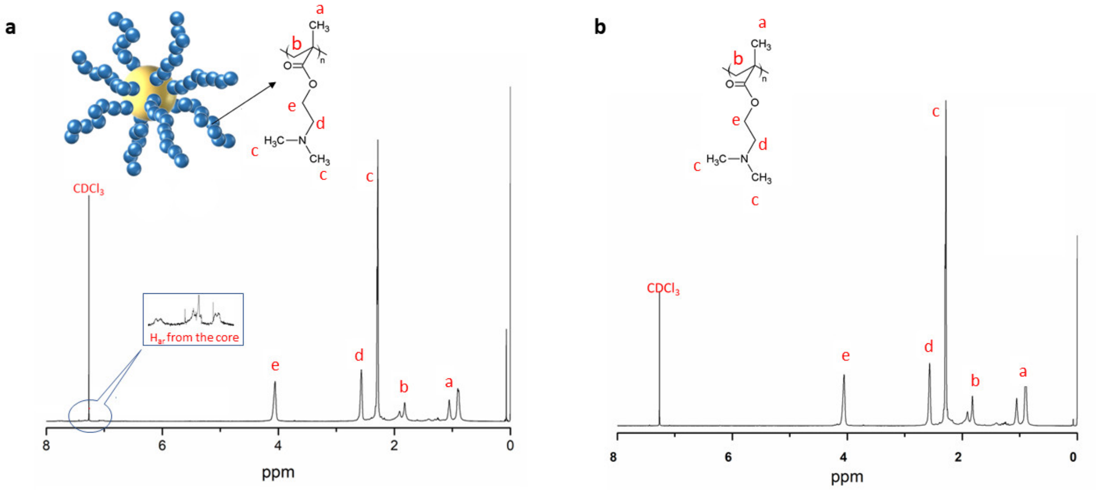

Figure S1. ^{1}H NMR spectra of (**a**) star PDMAEMA and (**b**) linear PDMAEMA ($CDCl_3$, 600 MHz)

The ^{1}H NMR spectra of both polymers (Figure S1a, b) displayed peaks for α-methyl groups and methylene groups in the methacrylate backbone at δ = 0.8–1.1 ppm (a) and 1.7–2.0 ppm (b), respectively. Proton signals from the methylene groups in pendant chains were found at δ = 2.5–2.6 ppm (d) and δ = 4.0–4.3 ppm (e), and signals from the methyl protons of the amino group were at δ = 2.2–2.4 ppm (c). Additionally, on star spectra are visible signals corresponding to the aromatic protons of the core in the range of 7.0–7.5 ppm (inset Figure S1a).

© 2020 by the authors. Submitted for possible open access publication under the terms and conditions of the Creative Commons Attribution (CC BY) license (http://creativecommons.org/licenses/by/4.0/).
